# High Prevalence of ESBL and Plasmid-Mediated Quinolone Resistance Genes in *Salmonella enterica* Isolated from Retail Meats and Slaughterhouses in Egypt

**DOI:** 10.3390/antibiotics10070881

**Published:** 2021-07-20

**Authors:** Wesam A. Adel, Ashraf M. Ahmed, Yamen Hegazy, Helmy A. Torky, Tadashi Shimamoto

**Affiliations:** 1 Department of Bacteriology, Mycology and Immunology, Faculty of Veterinary Medicine, Kafrelsheikh University, Kafr El-Sheikh 33516, Egypt; vet_wesamahmed105@yahoo.com; 2 Department of Animal Medicine, Faculty of Veterinary Medicine, Kafrelsheikh University, Kafr El-Sheikh 33516, Egypt; yamen_hegazy@vet.kfs.edu.eg; 3 Department of Microbiology, Faculty of Veterinary Medicine, Alexandria University, Alexandria 22758, Egypt; helmy.torky@alexu.edu.eg; 4 Laboratory of Food Microbiology and Hygiene, Graduate School of Integrated Sciences for Life, Hiroshima University, Higashihiroshima 739-8528, Japan

**Keywords:** antimicrobial resistance, Africa, AmpC, food safety, plasmids

## Abstract

The emergence and spread of multidrug-resistant *Salmonella enterica* (*S. enterica*) to humans through food of animal origin are considered a major global public health concern. Currently, little is known about the prevalence of important antimicrobial resistance genes in *S. enterica* from retail food in Africa. Therefore, the screening and characterization of the extended-spectrum β-lactamase (ESBL) and plasmid-mediated quinolone resistance (PMQR) genes in *S. enterica* isolated from retail meats and slaughterhouses in Egypt were done by using PCR and DNA sequencing techniques. Twenty-eight out of thirty-four (82.4%) non-duplicate *S. enterica* isolates showed multidrug-resistance phenotypes to at least three classes of antimicrobials, and fourteen (41.2%) exhibited an ESBL-resistance phenotype and harbored at least one ESBL-encoding gene. The identified β-lactamase-encoding genes included *bla*_CTX-M-1_, *bla*_CTX-M-3_, *bla*_CTX-M-13_, *bla*_CTX-M-14_, *bla*_CTX-M-15_, and *bla*_SHV-12_ (ESBL types); *bla*_CMY-2_ (AmpC type); and *bla*_TEM-1_ and *bla*_OXA-1_ (narrow-spectrum types). PMQR genes (included *qnrA*, *qnrB*, *qnrS*, and *aac(6′)-Ib-cr*) were identified in 23 (67.6%) isolates. The presence of ESBL- and PMQR-producing *S. enterica* with a high prevalence rate in retail meats and slaughterhouses is considered a major threat to public health as these strains with resistance genes could be transmitted to humans through the food chain.

## 1. Introduction

Food safety is a significant global public health concern. Unsafe food can lead to the transmission of a wide range of foodborne illnesses and outbreaks. According to a recent report from the WHO, an estimated 600 million (approximately 1 in 10 people worldwide) get ill after eating contaminated food and 420,000 die every year, resulting in the loss of 33 million disability-adjusted life years [1]. Currently, the recommended treatment options for salmonellosis include extended-spectrum cephalosporins and fluoroquinolones, as resistance to older antimicrobials (e.g., ampicillin, trimethoprim-sulfamethoxazole, and chloramphenicol) has been increasing for several years [2]. In recent years, the emergence of non-typhoidal *Salmonella enterica* with multidrug resistance to the extended-spectrum cephalosporins and fluoroquinolones has posed a serious global public health concern [3]. The resistance to cephalosporins and fluoroquinolones, as critically important antibiotics for human health, will lead to increased severity, morbidity, and mortality of salmonellosis in humans and consequently the use of last-line antimicrobials (e.g., carbapenems) [4]. In developing countries, this problem is intensified by the misuse and overuse of antimicrobial agents in humans, animals, poultry, and aquatic systems [5]. In many African countries, street food vending has become increasingly important to poorer economies; however, such foods are prepared under poor sanitation and unhygienic environments [6]. The majority of developed countries utilize a regular surveillance and monitoring system for antimicrobial drug resistance (AMR) that is updated regularly [7]. For example, the National Antimicrobial Resistance Monitoring Systems (NARMS) in the United States and the Danish Integrated Antimicrobial Resistance Monitoring and Research Programme (DANMAP) in Denmark [8,9]. Therefore, the AMR phenomenon is well mapped and monitored in these countries [10]. Alternatively, in developing countries, there is no regular surveillance or monitoring system for AMR, owing to the lack of surveillance networks, laboratory capacity, and appropriate diagnostics [11]. Recently, many studies have been conducted in both developed and developing countries to monitor the prevalence of ESBL-producing *S. enterica* in meat products, such as the United States [12], Italy [13], Chile [14], Bangladesh [15], and Brazil [16]. Currently, there is a considerable lack of information related to tracking and monitoring the emergence and incidence of antimicrobial resistance genes in pathogenic bacteria from food in Africa. Notably, between January and September 2010, we carried out the first large-scale survey in Africa to estimate the prevalence of antimicrobial resistance genes in *S. enterica* isolated from retail meat and dairy products in Egypt [17]. Therefore, the objectives of this study were to monitor the prevalence of extended-spectrum β-lactamase (ESBL) and plasmid-mediated quinolone resistance (PMQR) genes in *S. enterica* isolated from retail meats and slaughterhouses in Egypt and also to compare the change in the prevalence rates of ESBL and PMQR genes with those previously reported by our team ten years ago [17].

## 2. Results

### 2.1. Prevalence of MDR and ESBL-Producing S. enterica Isolated from Retail Meat and Beef Carcasses

**Figure 1:**
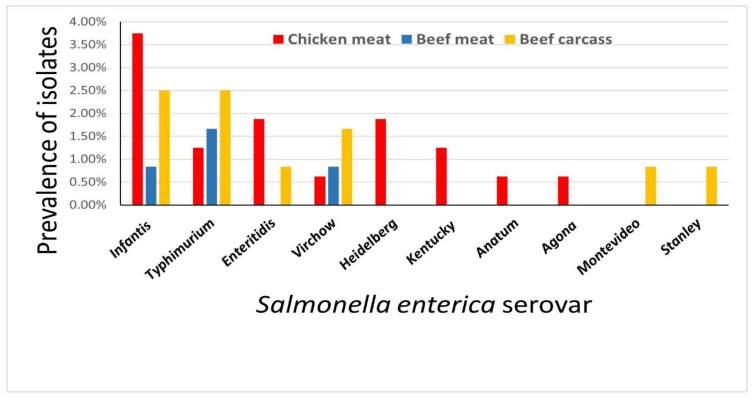
Prevalence of different *Salmonella enterica* serovars isolated from retail chicken meat, beef meat, and beef carcasses in Egypt.

**Figure 2:**
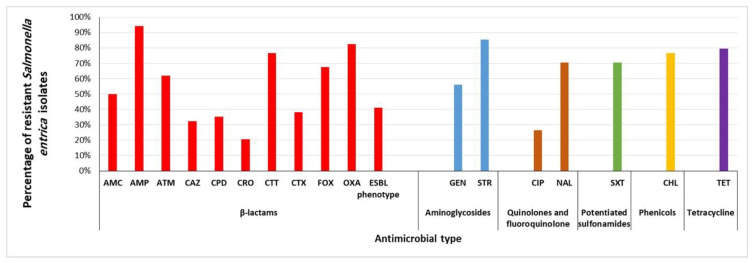
Resistance phenotypes of *Salmonella enterica* isolated from retail chicken meat, beef meat, and beef carcasses in Egypt. AMC, amoxicillin-clavulanic acid; AMP, ampicillin; ATM, aztreonam; CHL, chloramphenicol; CIP, ciprofloxacin; CAZ, ceftazidime; CPD, cefpodoxime; CRO, ceftriaxone; CTT, cefotetan; CTX, cefotaxime; ESBL, extended-spectrum β-lactamase; FOX, cefoxitin; GEN, gentamicin; NAL, nalidixic acid; OXA, oxacillin; STR, streptomycin; SXT, sulfamethoxazole-trimethoprim; TET, tetracycline.

In this study, non-duplicate isolates of *S. enterica* were detected in 34 (11.3%) of the 400 samples (chicken and beef meat and beef carcass swabs) analyzed. *S. enterica* isolates were serologically categorized into ten *S. enterica* serovars. (Figure 1, Supplementary Table S1). Twenty-eight out of 34 (82.4%) *S. enterica* isolates showed MDR phenotypes to at least three classes of antimicrobials. MDR was defined as isolates showing resistance to three or more antimicrobial classes [18]. The most prevalent resistance was to ampicillin, streptomycin, oxacillin, and tetracycline. Additionally, 14 (41.2%) of 34 *S. enterica* isolates showed ESBL-resistant phenotypes (Figure 2, Table 1, Supplementary Tables S2 and S3).

### 2.2. Prevalence of β-Lactamase-Encoding Genes in S. enterica Isolated from Retail Meat and Beef Carcasses in Egypt

PCR and DNA sequencing identified the CTX-M-encoding gene *bla*_CTX-M_ in 11 (32.4%) *S. enterica* isolates. The SHV-encoding gene *bla*_SHV-12_ was identified in 5 (14.7%) *S. enterica* isolates (Table 2). Additionally, the AmpC β-lactamase-encoding gene *bla*_CMY-2_ was identified in 9 isolates (26.5%) of *S. enterica* (Table 2). Finally, the narrow-spectrum β-lactamase-encoding genes *bla*_TEM-1_ and *bla*_OXA-1_ were identified in 27 (79.4%) and 10 (29.4%) *S. enterica* isolates, respectively (Table 2). Of note, the resistance phenotypes were expressed for all β-lactamase-encoding genes (Table 1).

### 2.3. Prevalence of Plasmid-Mediated Quinolone Resistance Genes in S. enterica Isolated from Retail Meats and Beef Carcasses in Egypt

Multiplex PCR screening identified plasmid-mediated quinolone resistance (PMQR) genes in 23 (67.6%) *S. enterica* isolates. The prevalence of PMQR genes was as follows: *qnrS* in 12 (35.3%) *S. enterica* isolates, *qnrB* in 8 (23.5%) *S. enterica* isolates, and *aac(6′)-Ib-cr* in 7 (23.5%) isolates (Table 1 and Table 2). Of note, all *qnr* containing isolates were resistant to nalidixic acid, and some of them were resistant to both nalidixic acid and ciprofloxacin (Table 1).

### 2.4. Comparison between the Prevalence Rates of Resistance Phenotypes and Genes in Salmonella Enterica Isolated from Retail Meats and Beef Carcasses in 2010 and 2020 in Egypt

**Figure 3:**
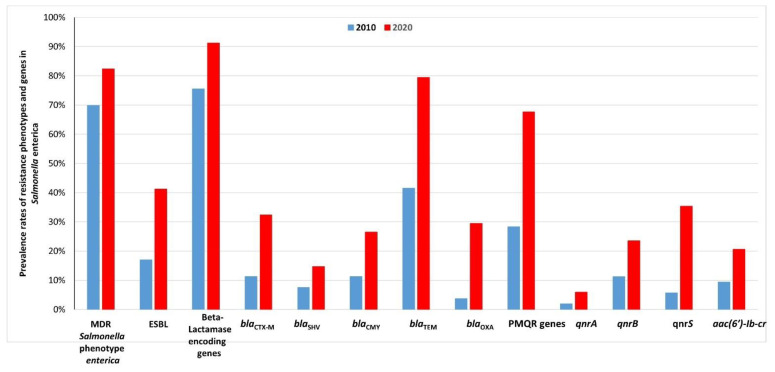
Comparison between the prevalence rates of resistance phenotypes and genes in *Salmonella enterica* isolated from retail meats and beef carcasses in 2010 and 2020 in Egypt.

Regarding a comparison between our results in the previous study in 2010 and the current study: MDR *S. enterica* was detected with prevalence rates: 69.8% and 82.4%, respectively; ESBL-resistant *S. enterica* with prevalence rates: 17% and 41.2%, respectively; β-lactamase-encoding genes with prevalence rates: 75.1% and 91.2%, respectively; and finally, PMQR genes with prevalence rates: 28.3% and 67.6%, respectively (Figure 3, Supplementary Table S4).

### 2.5. Transferability and Replicon Typing of Plasmids

PCR screening for replicon typing of plasmids revealed the presence of the following incompatibility groups: IncI1 in 9 (26.5%); IncA/C in 8 (23.5%); IncN in 7 (20.6%); IncHI1 in 5 (14.7%); IncHI1 in 4 (11.8%); and IncL/M in 1 (2.9%) *S. enterica* isolate (Supplementary Table S5). Moreover, the results of conjugation experiments showed that plasmids are conjugable in 26 (76.5%) *S. enterica* isolates, with most resistance genes transferred to the transconjugant *E. coli* HB101 (Supplementary Table S5).

## 3. Discussion

### 3.1. High Prevalence of MDR and ESBL-Producing S. enterica Isolated from Retail Meat and Beef Carcasses in Egypt

Antimicrobial resistance (AMR) is an increasingly growing problem that represents a threat to our capacity to treat common bacterial infections. AMR has recently been considered one of the top 10 global public health threats facing humanity according to WHO’s report [19]. The rapid and high global spread of multidrug-resistant bacteria that cause infections that cannot be treated with existing antimicrobial agents is particularly alarming [19]. The emergence and spread of multidrug-resistant (MDR) bacteria have led to the exacerbation of the AMR phenomenon worldwide due to the misuse and overuse of antimicrobials [1]. In the United States, at least 2 million people per annum acquire serious bacterial infections that are resistant to one or more of the antibiotics designed to treat those infections. Additionally, at least 23,000 people die annually as a direct result of these antibiotic-resistant infections [20]. Multidrug-resistant *S. enterica* spreads from animals to people predominantly through food [21]. In the USA, the CDC notes resistance to ceftriaxone and some level of resistance to ciprofloxacin in approximately 3% of non-typhoidal *S. enterica* tested. Approximately 5% of non-typhoidal *S. enterica* tested by the CDC are resistant to five or more types of drugs [20].

The prevalence of MDR *S. enterica* in meat products varies among different countries. In our study, MDR *S. enterica* was detected in 82.4% of retail meat samples. This prevalence rate is considerably higher compared to our previous report (69.8%) on MDR *S. enterica* in retail meats collected in 2010 in Egypt [17] (Figure 3, Supplementary Table S4) and also compared to that recently reported (68.5%) in China for MDR *S.* Enteritidis strains collected from retail foods in 39 cities [22] and that reported (50.9%) in South Korea for MDR *S. enterica* isolated from retail chicken meat [23]. However, it is relatively low compared to another recent study from Egypt, which showed all (100%) *S. enterica* serovars isolated from retail chickens were MDR [24]. Additionally, the prevalence of ESBL-resistant *S. enterica* isolates increased significantly from 17% in 2010 to 41.2% in the current study (Figure 3, Supplementary Table S3). Alternatively, our results are considered relatively low compared to that recently reported in chicken meat from Bangladesh as all (100%) *S. enterica* isolates were MDR and 58.1% of isolates were ESBL producers [15]. Additionally, a recent report from Italy showed that 80.5% of *S.* Infantis isolates from a broiler food chain exhibited ESBL phenotypes [13]. Similarly, 94% of *S.* Infantis isolates from chicken meat in Chile were MDR, and 63.2% were broad-spectrum β-lactam resistant [14]. Notably, raw chicken and sushi in Spain are the riskiest products in terms of transmission of ESBL-producing Enterobacteriaceae (occurrence 53.1% and 19.4%, respectively) [25]. Of note, our results are also considered relatively low compared to that we have recently reported from retail fishes in Egypt, as all (100%) *S*. *enterica* isolates were MDR and 57.9% of isolates were ESBL producers [26].

### 3.2. High Prevalence of β-Lactamase-Encoding Genes in S. enterica Isolated from Retail Meat and Beef Carcasses in Egypt

The production of β-lactamases is considered the main mechanism of resistance to β-lactam antibiotics in bacteria. The most common types of β-lactamases are categorized as follows: narrow-spectrum β-lactamases (TEM-1 and OXA-1) primarily confer resistance to first- and second-generation cephalosporins; AmpC β-lactamase (CMY) confers resistance to cephamycins (such as cefoxitin and cefotetan), and ESBLs (mainly CTX-M and SHV) confer resistance to expanded-spectrum cephalosporins, such as third- and fourth-generation cephalosporins [27,28]. The resistance of *S. enterica* to extended-spectrum cephalosporins is of major concern as these antibiotics are usually used as a front-line treatment for typhoid fever and other *S. enterica* infections in hospitals [29]. In this study, PCR and DNA sequencing showed that ESBL-producing *S. enterica* isolates carried at least one ESBL-encoding gene. These genes included *bla*_CTX-M_ (types 1, 2, 3, 13, 14, and 15) in *S. enterica* isolates (*S.* Typhimurium, *S.* Enteriditis, *S.* Infantis, *S.* Kentucky, and *S*. Virchow) and *bla*_SHV-12_ in *S. enterica* isolates (*S.* Typhimurium, *S.* Enteriditis, *S.* Infantis, *S.* Kentucky and *S*. Heidelberg). There was a significant increase in the prevalence rates of *bla*_CTX-M_ (32.4%) and *bla*_SHV-12_ (14.7%) recorded in this study and those of *bla*_CTX-M_ (11.3%) and *bla*_SHV-12_ (7.5%) previously reported in retail meats collected in 2010 in Egypt [17] (Figure 3, Supplementary Table S4). Recently, 2.7% of *S. enterica* isolates in chicken meat in Bangladesh were positive for *bla*_CTX-M-1_ [15]. Similarly, *bla*_CTX-M_ (3.2%) and *bla*_SHV_ (4.8%) were detected in *S.* Heidelberg strains from the poultry production chain (poultry, poultry meat, and a poultry farm) in Brazil [16]. Furthermore, a recent report from Italy showed that 80.5% of *S.* Infantis isolates from a broiler food chain possessed the *bla*_CTX-M-1_ gene [13]. In the USA, 61% of *S.* Infantis isolates from the poultry production chain carried the *bla*_CTX-M-65_ gene [12]. In Russia, all MDR *S*. Infantis isolates from chicken food products carried the *bla*_CTX-M-14_ gene [30]. In South Korea, all ESBL-resistant *S.* Virchow in chicken carcass samples were positive for *bla*_CTX-M-15_ [31], and only 31.0% of ESBL-resistant *S.* Virchow and *S.* Enteritidis isolated from retail chicken meat were positive for *bla*_CTX-M-15_ and *bla*_CTX-M-79_ [23]. In China, the *bla*_CTX-M-14_ gene was found in an MDR *S.* Kentucky strain isolated from a poultry slaughterhouse [32], and more recently, the *bla*_CTX-M-55_ gene was detected in 2.4% of *S.* Enteritidis strains isolated from retail foods [22]. More recently, in the Thailand, Cambodia, Lao PDR, and Myanmar border area, the *bla*_CTX-M-55_ and *bla*_CTX-M-14_ genes were identified in ESBL-producing *S. enterica* (1.9%) and *E. coli* (6.3%) strains isolated from pigs and pork [33]. Notably, the predominant ESBL-encoding genes in Enterobacteriaceae isolated from raw chicken and sushi in Spain were *bla*_SHV-12_ (50.1%), and *bla*_CTX-M_ (20.8%) [25]. Additionally, more recently, we have identified *bla*_CTX-M-3_, *bla*_CTX-M-14_, *bla*_CTX-M-15_, *bla*_SHV-1_, *bla*_SHV-2_, and *bla*_SHV-12_ in *S. enterica* isolated from retail fishes in Egypt [26]. Interestingly, recently in Japan, ESBL-producing *S*. enterica isolates carrying *bla*_CTX-M-15_ or *bla*_CTX-M-14_ genes were identified in the stool samples of healthy food workers from several restaurants and food factories [34]. Therefore, food workers act as a reservoir of ESBL-producing *S. enterica* and may contribute to the spread of these dangerous bacteria via food products of animal origin. It is worth mentioning that, more recently in Peru, an MDR *S.* Infantis clone carrying *bla*_CTX-M-65_ and causing diarrhea in children has been disseminated between retail chicken meat and children [35]. Therefore, there is also a possibility of the dissemination of MDR *S.* enterica strains (carrying ESBL-and PMQR genes) between retail meats and humans in Egypt, and therefore, it is considered a major threat to public health.

### 3.3. High Prevalence of Plasmid-Mediated Quinolone Resistance Genes in S. enterica Isolated from Retail Meats and Beef Carcasses in Egypt

Fluoroquinolones are widely used in human and veterinary practices worldwide to treat bacterial diseases. Therefore, *S. enterica* with resistance or reduced susceptibility to fluoroquinolones is of serious concern, as these compounds are among the first choice of antimicrobials for the treatment of invasive and systemic salmonellosis in humans and animals [36,37]. The plasmid-mediated quinolone resistance (PMQR) genes confer decreased susceptibility to fluoroquinolones and enhance a high level of fluoroquinolone resistance in association with chromosomal mutations in DNA gyrase and topoisomerase IV genes [38]. The most common PMQR genes among Gram-negative bacteria are *qnrA*, *qnrB*, *qnrS*, and *aac(6′)-Ib-cr* [38]. The contribution of plasmid-mediated quinolone resistance (PMQR) genes in the emergence and spread of fluoroquinolones resistance among *S. enterica* is well-documented worldwide. In our study, the prevalence of PMQR genes from retail meats was significantly higher (67.6%) than that (28.3%) reported from our previous study on *S. enterica* isolates collected in 2010 from Egypt [17] (Figure 3, Supplementary Table S3). In the current study, PMQR genes: *qnrA*, *qnrB*, *qnrS*, and *aac(6′)-Ib-cr* were found at high prevalence rates: 5.9%, 23.5%, 35.3%, and 20.6%, respectively, compared with low prevalence rates: 1.9%, 11.2%, 5.7%, and 20.6%, respectively, reported in our previous study [17] (Figure 3, Supplementary Table S3). Notably, the occurrence of PMQR genes in *S. enterica* isolates from retail meats varies greatly worldwide. In Bangladesh, the prevalence of *qnrA* and *qnrS* genes in *S. enterica* from chicken meat was 4.1 and 6.8%, respectively [15]. In Chile, *qnrB* was detected in 2.3% of *S.* Infantis isolates from chicken meat [14]. In the USA, there was a significantly high prevalence rate (90%) of PMQR genes in *S. enterica* isolates from swine cecal contents and retail pork products, and the prevalence rates of *qnrB* and *qnrS* genes were 80% and 6.7%, respectively [39]. More recently, in China, the *qnrS* gene was detected in 0.8% of *S.* Enteritidis strains isolated from retail foods [22].

### 3.4. Common Plasmid Replicon Types in S. enterica Isolated from Retail Meat and Beef Carcasses in Egypt

Plasmids play an important role in the horizontal transfer of antimicrobial-resistance genes in Gram-negative bacteria including *S. enterica*. In our study, molecular analysis of plasmid transferability and replicon typing indicated that most plasmids (including ESBL-encoding genes and PMQR genes) are transferrable. PCR-based replicon typing showed that IncI1, IncA/C, IncN, IncHI1, and IncHI2 were the most common incompatibility groups (Supplementary Table S5). These incompatibility-group types were previously identified in plasmids among different ESBL and PMQR-producing *S. enterica* worldwide [17,26,40,41,42,43]. It is well known that these plasmid-incompatibility groups play a crucial role in the spread and dissemination of many antimicrobial resistance genes, particularly ESBL-encoding genes in Gram-negative bacteria [44]. Notably, in the USA, the IncI1 and IncA/C plasmids were responsible for the transfer and dissemination of the extended-spectrum cephalosporin resistance among *S.* Heidelberg from chicken meat to humans [45].

## 4. Materials and Methods

### 4.1. Sample Collection

A total of 400 samples (160 chicken meat, 120 beef meat, and 120 beef carcass swabs) were randomly collected from different street vendors, butchers, retail markets, and slaughterhouses in 38 cities from four governorates (Dakahlia, Damietta, Gharbia, and Kafr El-Sheikh) in Egypt, between January and September 2020 (details of samples sources and locations are present in Supplementary Tables S6 and S7). Samples were collected in sterile bags and labeled, then transferred in boxes with ice and examined immediately after arrival at the laboratory.

### 4.2. Isolation and Identification of S. enterica

*Salmonella* isolation was carried out by a standard cultivation method as recommended by ISO 6579-1 [46]. Samples (25 g meat or a swab pre-moistened with 25 mL buffered peptone water, Oxoid, UK) were inserted in stomacher bags containing buffered peptone water (225 mL). The homogenization was carried out at 320 rpm for 2 min, followed by incubation at 37 °C overnight. Then, 0.1 mL aliquots were inoculated into tubes containing 10 mL Rappaport Vassiliadis (RV) broth (Oxoid, UK) and then, incubated at 42 °C for 48 h. Then, XLD (xylose lysine deoxycholate) agar (Oxoid, UK) plates were inoculated from each of the RV broths and incubated at 37 °C for 18–24 h. Suspect colonies of *Salmonella* were biochemically confirmed using the API 20E system (bioMérieux, Marcy-l’Étoile, France). Then, *Salmonella* isolates were serotyped by using specific *Salmonella* O and H agglutinating antisera (Difco, Sparks, MD, USA) according to the Kauffman–White serotyping scheme [47]. Of note, local *S. enterica* strains isolated from retail meat and dairy products in Egypt were used as controls for the experiments [17].

### 4.3. Antimicrobial Sensitivity Testing and ESBL-Resistance Phenotyping

The Kirby–Bauer disk diffusion assay was used for the determination of antimicrobial-sensitivity phenotypes of bacterial isolates according to the standards and interpretive criteria described by the Clinical and Laboratory Standards Institute [48]. The types of antibiotics used are present in Figure 2. The disks were purchased from Oxoid, UK, and the results were recorded based on CLSI guidelines [48]. *Escherichia coli* ATCC 25922 was used as quality control. For the detection of the ESBL-resistance phenotype, the double-disc synergy test was used. Briefly, pairs of disks containing ceftazidime (CAZ), 30 µg, and cefotaxime (CTX), 30 µg, were used with and without amoxicillin–clavulanic acid (AMC) 20/10 μg on the same inoculated plate containing Muller-Hinton agar (Oxoid, UK). A positive test result was defined as a 5 mm increase in the zone diameter compared to that of a disk without clavulanic acid [49].

### 4.4. Preparation of Salmonella DNA

*Salmonella* DNA was prepared using boiled lysates, as previously described [17]. *Salmonella* colonies were subcultured in LB broth. Then, 200 μL of overnight bacterial culture was mixed with 800 μL of distilled water and boiled for 10 min. The resulting solution was centrifuged, and the supernatant was used as the DNA template and stored until use at −20 °C.

### 4.5. PCR and DNA Sequencing for β-Lactamase-Encoding Genes and Plasmid-Mediated Quinolone Resistance Genes

Screening for TEM, SHV, CTX-M, OXA, and CMY β-lactamase-encoding genes was performed by PCR using universal primers for the TEM, SHV, OXA, CTX-M, and CMY families, as described previously [50]. Screening for IMP, NDM, SPM, VIM, and OXA-48 carbapenemase-encoding genes was performed using multiplex PCR as described previously [51]. Finally, PCR amplification was used to screen for plasmid-mediated quinolone-resistance genes, *qnrA*, *qnrB*, *qnrS* and *aac(6′)-Ib-cr*, using previously described primers [50]. The PCR amplicons were subjected to electrophoresis in a 1.0% agarose gel, stained with ethidium bromide, and visualized under ultraviolet light. Then, PCR fragments were purified using a QIAquick Gel Extraction Kit (Qiagen, Tokyo, Japan) from the agarose gel. An ABI automatic DNA sequencer (Model 373; Perkin-Elmer, Waltham, MA, USA) was used for sequencing both strands of the PCR products. Primers are compiled in Supplementary Table S8. Local *S. enterica* strains isolated from retail meat and dairy products in Egypt and carrying resistance genes were used as controls for the experiments [17].

### 4.6. Plasmid Incompatibility Grouping and Transconjugation Experiments

The mating-out assay was used for the determination of the transferability of plasmids using *S. enterica* isolates as donors and a rifampicin-resistant mutant of *E. coli* HB101 as the recipient, as described previously [17]. Transconjugants were selected on agar supplemented with AMP (100 mg/L) and rifampicin (250 mg/L). Plasmid DNA was extracted from both *S. enterica* isolates and *E. coli* transconjugants using the Kado and Liu method [52]. PCR-based replicon typing was used for the determination of plasmid incompatibility grouping, as previously described [53]. PCR assays on the transconjugants were used for the confirmation of the transfer of resistance genes, as described previously [17]. Primers are compiled in Supplementary Table S8. Local *S. enterica* strains isolated from retail meat and dairy products in Egypt and carrying plasmid replicon types were used as controls for the experiments [17].

### 4.7. BLAST Analysis of the Sequence Data

The BLAST program (available at the NCBI BLAST homepage: http://blast.ncbi.nlm.nih.gov/Blast.cgi; accessed on 19 July 2021) was used for carrying out a similarity search for DNA sequencing data.

## 5. Conclusions

Our study highlights the role of retail meats as a potential source for MDR *S. enterica* strains carrying ESBL and PMQR genes. This is considered a potential public health threat that requires urgent attention from health professionals to ensure food safety in Egypt. Additionally, these findings emphasize the importance of continuous monitoring to track the emergence and changes in antibiotic resistance in the food chain in Egypt by ongoing surveillance in the future.

## Figures and Tables

**Table 1:** Resistance phenotypes and incidence of resistance genes in *Salmonella enterica* isolated from retail meats and beef carcasses in Egypt.

No.	Isolate	Serovar	Source	Resistance Phenotype	ESBL Phenotype	Resistance Gene(s)
1	SI-CM1	*S.* Infantis	Chicken meat	AMC, AMP, ATM, CAZ, CHL, CIP, CPD, CRO, CTT, CTX, FOX, GEN, NAL, OXA, STR, SXT, TET	+	*bla*_TEM-1_, *bla*_CTX-M-1_, *bla*_CMY-2_, *bla*_OXA-1_, *qnrB*, *aac(6′)-Ib-cr*
2	SI-CM2	*S.* Infantis	Chicken meat	AMC, AMP, ATM, CHL, CPD, CTT, CTX, FOX, GEN, OXA, STR, SXT, TET	*+*	*bla*_TEM-1_, *bla*_SHV-12_
3	SI-CM3	*S.* Infantis	Chicken meat	AMC, AMP, ATM, CAZ, CHL, CPD, CTT, CTX, FOX, GEN, OXA, STR, SXT, TET	*+*	*bla*_TEM-1_, *bla*_CTX-M-14_, *bla*_CMY-2_
4	SI-CM4	*S.* Infantis	Chicken meat	AMP, CHL, CTT, FOX, OXA, STR, TET	*-*	*bla*_OXA-1_
5	SI-CM5	*S.* Infantis	Chicken meat	AMP, CHL, CIP, NAL, STR, TET	*-*	*bla*_TEM-1_, *qnrS*
6	SI-CM6	*S.* Infantis	Chicken meat	AMC, AMP, ATM, CTT, FOX, GEN, OXA, STR, SXT, TET	*-*	*bla*_CMY-2_
7	SI-BM1	*S.* Infantis	Beef meat	AMP, ATM, CTT, FOX, OXA, STR, SXT, TET	*-*	*bla*_TEM-1_
8	SI-BC1	*S.* Infantis	Beef carcass	AMP, CHL, NAL, STR	*-*	*bla*_TEM-1_, *qnrB*
9	SI-BC2	*S.* Infantis	Beef carcass	AMP, CTT, FOX, OXA, STR	*-*	*bla*_OXA-1_
10	SI-BC3	*S.* Infantis	Beef carcass	AMP, NAL	*-*	*bla*_TEM-1_, *qnrS*
11	ST-CM1	*S.* Typhimurium	Chicken meat	AMC, AMP, ATM, CAZ, CHL, CIP, CPD, CRO, CTT, CTX, FOX, GEN, NAL, OXA, STR, SXT, TET	*+*	*bla*_TEM-1_, *bla*_CTX-M-15_, *bla*_CMY-2_, *bla*_OXA-1_, *qnrB*, *aac(6′)-Ib-cr*
12	ST-CM2	*S.* Typhimurium	Chicken meat	AMC, AMP, ATM, CAZ, CHL, CIP, CPD, CTT, CTX, FOX, GEN, NAL, OXA, STR, SXT, TET	*+*	*bla*_TEM-1_, *bla*_CTX-M-3_, *bla*_SHV-12_, *qnrB*
13	ST-BM1	*S.* Typhimurium	Beef meat	AMC, AMP, ATM, CHL, CTT, FOX, OXA, STR, SXT, TET	*-*	*bla*_TEM-1_
14	ST-BM2	*S.* Typhimurium	Beef meat	AMC, AMP, ATM, CAZ, CHL, CIP, CPD, CRO, CTT, CTX, FOX, GEN, NAL, OXA, STR, SXT, TET	*+*	*bla*_TEM-1_, *bla*_CTX-M-14_, *bla*_OXA-1_, *qnrS*
15	ST-BC1	*S.* Typhimurium	Beef carcass	AMC, AMP, ATM, CAZ, CHL, CPD, CTT, CTX, FOX, GEN, NAL, OXA, STR, SXT, TET	*+*	*bla*_TEM-1_, *bla*_CTX-M-13_, *qnrS*
16	ST-BC2	*S.* Typhimurium	Beef carcass	AMP, OXA, CHL, NAL, STR	*-*	*bla*_TEM-1_, *qnrA*
17	ST-BC3	*S.* Typhimurium	Beef carcass	AMP, ATM, CHL, CTT, FOX, GEN, NAL, OXA, STR, SXT, TET	*-*	*bla*_TEM-1_, *qnrS*
18	SE-CM1	*S.* Enteritidis	Chicken meat	AMC, AMP, ATM, CAZ, CHL, CIP, CPD, CRO, CTT, CTX, FOX, GEN, NAL, OXA, STR, SXT, TET	*+*	*bla*_TEM-1_, *bla*_CTX-M-3_, *bla*_CMY-2_, *bla*_OXA-1_, *qnrB*, *aac(6′)-Ib-cr*
19	SE-CM2	*S.* Enteritidis	Chicken meat	AMC, AMP, ATM, CAZ, CHL, CPD, CTT, CTX, FOX, GEN, NAL, OXA, STR, SXT, TET	*+*	*bla*_TEM-1_, *bla*_CTX-M-15_, *qnrS*
20	SE-CM3	*S.* Enteritidis	Chicken meat	AMC, AMP, ATM, CHL, CTT, CTX, FOX, NAL, OXA, STR, SXT TET	*+*	*bla*_TEM-1_, *bla*_SHV-12_, *qnrS*
21	SE-BC1	*S.* Enteritidis	Beef carcass	AMP, ATM, CTT, FOX, OXA, STR	*-*	*bla*_OXA-1_, *bla*_CMY-2_
22	SV-CM1	*S.* Virchow	Chicken meat	AMC, AMP, ATM, CAZ, CHL, CPD, CRO, CTT, CTX, FOX, GEN, NAL, OXA, STR, SXT, TET	*+*	*bla*_TEM-1_, *bla*_CTX-M-15_, *aac(6′)-Ib-cr*
23	SV-BM1	*S.* Virchow	Beef meat	AMP, CHL, CTT, GEN, NAL, OXA, STR, SXT, TET	*-*	*bla*_TEM-1_, *qnrS*
24	SV-BC1	*S.* Virchow	Beef carcass	AMC, AMP, ATM, CHL, CTT, FOX, GEN, OXA, STR, SXT, TET	*-*	*bla*_TEM-1_, *bla*_OXA-1_, *bla*_CMY-2_
25	SV-BC2	*S.* Virchow	Beef carcass	AMP, NAL, CHL	*-*	*qnrB*
26	SH-CM1	*S*. Heidelberg	Chicken meat	AMC, AMP, ATM, CAZ, CHL, CIP, CPD, CRO, CTT, FOX, GEN, NAL, OXA, STR, SXT, TET	*+*	*bla*_TEM-1_, *bla*_CTX-M-2_, *bla*_CMY-2_, *bla*_SHV-12_, *qnrB*
27	SH-CM2	*S*. Heidelberg	Chicken meat	AMP, ATM, CHL, CTT, FOX, GEN, OXA, STR, SXT, TET	*-*	*bla*_TEM-1_, *bla*_CMY-2_
28	SH-CM3	*S*. Heidelberg	Chicken meat	AMP, OXA, NAL, TET	*-*	*bla*_TEM-1_, *qnrB*
29	SK-CM1	*S.* Kentucky	Chicken meat	AMC, AMP, CAZ, CHL, CIP, CPD, CRO, CTT, CTX, FOX, GEN, NAL, OXA, STR, SXT, TET	*+*	*bla*_TEM-1_, *bla*_CTX-M-15_, *bla*_CMY-2_*, bla*_OXA-1_, *qnrS*, *aac(6′)-Ib-cr*
30	SK-CM2	*S.* Kentucky	Chicken meat	AMC, AMP, ATM, CHL, CIP, CTT, CTX, FOX, GEN, NAL, OXA, STR, SXT, TET	*+*	*bla*_TEM-1_, *bla*_SHV-12_, *aac(6′)-Ib-cr*
31	SAN-CM1	*S*. Anatum	Chicken meat	AMP, CHL, CTT, NAL, OXA, STR, SXT, TET	*-*	*bla*_OXA-1_, *qnrS*
32	SAG-CM1	*S*. Agona	Chicken meat	NAL	*-*	*qnrA*
33	SM-BC1	*S*. Montevideo	Beef carcass	AMP, ATM, CHL, CTT, GEN, NAL, OXA, STR, SXT, TET	*-*	*bla*_TEM-1_, *qnrS*
34	SS-BC1	*S.* Stanley	Beef carcass	NAL, TET	*-*	*qnrS*

**Table 2:** Prevalence of resistance genes in *Salmonella enterica* serovars isolated from retail meats and beef carcasses in Egypt.

*S. enterica* Serovar	B-Lactamases Resistance Genes					Plasmid-Mediated Quinolone Resistance Genes			
	ESBL-Type (No.)		Narrow-Spectrum Types (No.)		AmpC (*bla*_CMY_) (No.)	*qnrA*	*qnrB*	*qnrS*	*aac(6′)-Ib-cr*
	*bla*_CTX-M_	*bla*_SHV_	*bla*_TEM_	*bla*_OXA_					
Infantis	*bla*_CTX-M-1_ (1) *bla*_CTX-M-14_ (1)	*bla*_SHV-12_ (1)	*bla*_TEM-1_ (7)	*bla*_OXA-1_ (3)	*bla*_CMY-2_ (3)	-	2	2	1
Typhimurium	*bla*_CTX-M-3_ (1) *bla*_CTX-M-13_ (1) *bla*_CTX-M-__14_(1) *bla*_CTX-M-15_ (1)	*bla*_SHV-12_ (1)	*bla*_TEM-1_ (7)	*bla*_OXA-1_ (2)	*bla*_CMY-2_ (1)	1	2	3	2
Enteritidis	*bla*_CTX-M-3_ (1) *bla*_CTX-M-15_ (1)	*bla*_SHV-12_ (1)	*bla*_TEM-1_ (3)	*bla*_OXA-1_ (2)	*bla*_CMY-2_ (1)	-	1	2	1
Virchow	*bla*_CTX-M-15_ (1)	-	*bla*_TEM-1_ (3)	*bla*_OXA-1_ (1)	*bla*_CMY-2_ (1)	-	1	1	1
Heidelberg	*bla*_CTX-M-2_ (1)	*bla*_SHV-12_ (1)	*bla*_TEM-1_ (3)	-	*bla*_CMY-2_ (2)	-	2	-	-
Kentucky	*bla*_CTX-M-15_ (1)	*bla*_SHV-12_ (1)	*bla*_TEM-1_ (2)	*bla*_OXA-1_ (1)	*bla*_CMY-2_ (1)	-	-	1	2
Anatum	-	-	-	*bla*_OXA-1_ (1)	-	-	-	1	-
Agona	-	-	-	-	-	1	-	-	-
Montevideo	-	-	*bla*_TEM-1_ (2)	-	-	-	-	1	-
Stanley	-	-	-	-	-	-	-	1	-
Total	11 (32.4%)	5 (14.7%)	27 (79.4%)	10 (29.4%)	9 (26.5%)	2 (5.9%)	8 (23.5%)	12 (35.3%)	7 (20.6%)

## Data Availability

The authors confirm that the data that support the findings of this study are available within the article.

## Supplementary Materials

The following are available online at https://www.mdpi.com/article/10.3390/antibiotics10070881/s1 Table S1: Prevalence of *Salmonella enterica* serovars isolated from retail meats and slaughterhouses. Table S2: Resistance phenotypes of *Salmonella enterica* isolated from retail meats and slaughterhouses. Table S3: The sources of resistance phenotypes of *Salmonella enterica* isolated from retail meats and beef carcasses in Egypt. Table S4: Comparison between the prevalence rates of resistance phenotypes and genes in *Salmonella enterica* isolated from retail meats and slaughterhouses in 2010 and 2020 from Egypt. Table S5: Results of conjugation experiments and plasmid replicon typing for *Salmonella enterica* isolated from retail meats and beef carcasses in Egypt. Table S6: Numbers and sources of meat samples used in this study. Table S7: City names and numbers of meat samples collected from four governorates (Dakahlia, Damietta, Gharbia, and Kafr El-Sheikh) in Egypt. Table S8: Primers used for PCR and DNA sequencing.
