# Supplementary material for: High Prevalence of ESBL and Plasmid-Mediated Quinolone Resistance Genes in Salmonella enterica Isolated from Retail Meats and Slaughterhouses in Egypt
Source: Antibiotics (Basel). 2021 Jul 20;10(7):881. doi: 10.3390/antibiotics10070881 (PMC8300843; doi:10.3390/antibiotics10070881)
Supplement: Supplementary file 1 [file antibiotics-10-00881-s001.zip › antibiotics-1289009-supplementary.pdf]

Table S1. Prevalence of *Salmonella enterica serovars* isolated from retail meats and beef carcasses in Egypt

S. enterica serovar	Samples			Total (400)
	Chicken meat (160)	Beef meat (120)	Beef carcass (120)	
Infantis	6 (3.75%)	1 (0.83%)	3 (2.50%)	10 (2.5%)
Typhimurium	2 (1.25%)	2 (1.67%)	3 (2.50%)	7 (1.8%)
Enteritidis	3 (1.88%)	0.00%	1 (0.83%)	4 (1%)
Virchow	1 (0.63%)	1 (0.83%)	2 (1.67%)	4 (1%)
Heidelberg	3 (1.88%)	0.00%	0.00%	3 (0.8%)
Kentucky	2 (1.25%)	0.00%	0.00%	2 (0.5%)
Anatum	1 (0.63%)	0.00%	0.00%	1 (0.3%)
Agona	1 (0.63%)	0.00%	0.00%	1 (0.3%)
Montevideo	0.00%	0.00%	1 (0.83%)	1 (0.3%)
Stanley	0.00%	0.00%	1 (0.83%)	1 (0.3%)
Total	19 (11.88)	4 (3.33%)	11 (9.17)	34 (8.5%)

Table S2. Resistance phenotypes of *Salmonella enterica* isolated from retail meats and beef carcasses in Egypt

Antimicrobials tested[a]	Number (%) of resistant isolates *Salmonella* (n=34)
β-lactams	
AMC	17 (50%)
AMP	32 (94.1%)
ATM	21 (61.8%)
CAZ	11 (32.4%)
CPD	12 (35.3%)
CRO	7 (20.6%)
CTT	26 (76.5%)
CTX	13 (38.2%)
FOX	23 (67.6%)
OXA	28 (82.4%)
ESBL phenotype	14 (41.2%)
Aminoglycosides	
GEN	19 (55.9%)
STR	29 (85.3%)
Quinolones and fluoroquinolone	
CIP	9 (26.5%)
NAL	24 (70.5%)
Potentiated sulfonamides	
SXT	24 (70.5%)
Phenicols	
CHL	26 (76.5%)
Tetracycline	
TET	27 (79.4%)

[a] AMC, amoxicillin-clavulanic acid; AMP, ampicillin; ATM, aztreonam; CHL, chloramphenicol; CIP, ciprofloxacin; CAZ, ceftazidime; CPD, cefpodoxime; CRO, ceftriaxone; CTT, cefotetan; CTX, cefotaxime; ESBL, extended-spectrum β-lactamase; FOX, cefoxitin; GEN, gentamicin; NAL, nalidixic acid; OXA, oxacillin; STR, streptomycin; SXT, sulfamethoxazole-trimethoprim; TET, tetracycline.

Table S3. The sources of resistance phenotypes of *Salmonella enterica* isolated from retail meats and beef carcasses in Egypt

Antimicrobials tested	Number (%) of resistant isolates[a]			Number (%) of resistant isolates (n=34)
	Chicken meat (n=19)	Beef meat (n=4)	Beef carcass (n=11)	
β-lactams				
AMC	13 (68.4%)	2 (50%)	2 (18.2%)	17 (50%)
AMP	18 (94.7%)	4 (100%)	10 (90.9%)	32 (94.1%)
ATM	13 (68.4%)	3 (75%)	5 (45.5%)	21 (61.8%)
CAZ	9 (47.4%)	1 (25%)	1 (9.1%)	11 (32.4%)
CPD	10 (52.6%)	1 (25%)	1 (9.1%)	12 (35.3%)
CRO	6 (31.6%)	1 (25%)	0 (0%)	7 (20.6%)
CTT	16 (84.2%)	4 (100%)	6 (54.5%)	26 (76.5%)
CTX	11 (57.9%)	1 (25%)	1 (9.1%)	13 (38.2%)
FOX	15 (78.9%)	3 (75%)	5 (45.5%)	23 (67.6%)
OXA	17 (89.5%)	4 (100%)	7	28 (82.4%)
ESBL phenotype	12 (63.2%)	1 (25%)	1 (9.1%)	14 (41.2%)
Aminoglycosides				
GEN	13 (68.4)	2 (50%)	4 (36.4%)	19 (55.9%)
STR	17 (89.5%)	4 (100%)	8 (72.7%)	29 (85.3%)
Quinolones and fluoroquinolone				
CIP	8 (42.1%)	1 (25%)	0 (0%)	9 (26.5%)
NAL	14 (73.7%)	2 (50%)	8 (72.7%)	24 (70.5%)
Potentiated sulfonamides				
SXT	15 (78.9%)	4 (100%)	4 (36.4%)	24 (70.5%)
Phenicols				
CHL	16 (84.2%)	3 (75%)	7 (63.6%)	26 (76.5%)
Tetracycline				
TET	17 (89.5%)	4 (100%)	6 (54.5%)	27 (79.4%)

[a] n= Number of *S. enterica* isolates

Table S4. Comparison between the prevalence rates of resistance phenotypes and genes in *Salmonella* enterica isolated from retail meats and beef carcasses in 2010 and 2020 from Egypt

Criterion[a]	2010	2020
MDR *Salmonella enterica*	69.8%	82.4%
ESBL phenotype	17%	41.2%
Beta-Lactamase-encoding genes	75.5%	91.2%
bla_{CTX-M}	11.3%	32.4%
bla_{SHV}	7.5%	14.7%
bla_{CMY}	11.3%	26.5%
bla_{TEM}	41.5%	79.4
bla_{OXA}	3.7%	29.4%
PMQR genes	28.3%	67.6%
qnrA	1.9%	5.9%
qnrB	11.2%	23.5%
qnrS	5.7%	35.3%
aac(6')-Ib-cr	9.4%	20.6%

[a] MDR, multidrug-resistant; ESBL, extended-spectrum β-lactamase; PMQR, plasmid-mediated quinolone resistance

Table S5. Results of conjugation experiments and plasmid replicon typing for *Salmonella enterica* isolated from retail meats and beef carcasses in Egypt.

No.	Isolate	Serovar	Resistance gene(s)	Plasmid replicon	Conjugable	Transconjugant resistance genotype
1	SI-CM1	*S.* Infantis	$bla_{\text{TEM-1}}$, $bla_{\text{CTX-M-1}}$, $bla_{\text{CMY-2}}$, $bla_{\text{OXA-1}}$, *qnrB*, *aac(6′)-Ib-cr*	I1	Yes	$bla_{\text{TEM-1}}$, $bla_{\text{CTX-M-1}}$, $bla_{\text{CMY-2}}$, *qnrB*, *aac(6′)-Ib-cr*
2	SI-CM2	*S.* Infantis	$bla_{\text{TEM-1}}$, $bla_{\text{SHV-12}}$	HI1	Yes	$bla_{\text{TEM-1}}$, $bla_{\text{SHV-12}}$
3	SI-CM3	*S.* Infantis	$bla_{\text{TEM-1}}$, $bla_{\text{CTX-M-14}}$, $bla_{\text{CMY-2}}$	I1	Yes	$bla_{\text{TEM-1}}$, $bla_{\text{CTX-M-14}}$, $bla_{\text{CMY-2}}$
4	SI-CM4	*S.* Infantis	$bla_{\text{OXA-1}}$	I1	No	-
5	SI-CM5	*S.* Infantis	$bla_{\text{TEM-1}}$, *qnrS*	HI2	Yes	$bla_{\text{TEM-1}}$, *qnrS*
6	SI-CM6	*S.* Infantis	$bla_{\text{CMY-2}}$	I1	No	-
7	SI-BM1	*S.* Infantis	$bla_{\text{TEM-1}}$	A/C	Yes	$bla_{\text{TEM-1}}$
8	SI-BC1	*S.* Infantis	$bla_{\text{TEM-1}}$, *qnrB*	N	Yes	$bla_{\text{TEM-1}}$, *qnrB*
9	SI-BC2	*S.* Infantis	$bla_{\text{OXA-1}}$	A/C	No	-
10	SI-BC3	*S.* Infantis	$bla_{\text{TEM-1}}$, *qnrS*	HI1	Yes	$bla_{\text{TEM-1}}$, *qnrS*
11	ST-CM1	*S.* Typhimurium	$bla_{\text{TEM-1}}$, $bla_{\text{CTX-M-15}}$, $bla_{\text{CMY-2}}$, $bla_{\text{OXA-1}}$, *qnrB*, *aac(6′)-Ib-cr*	A/C	Yes	$bla_{\text{TEM-1}}$, $bla_{\text{CTX-M-15}}$, $bla_{\text{CMY-2}}$, *aac(6′)-Ib-cr*
12	ST-CM2	*S.* Typhimurium	$bla_{\text{TEM-1}}$, $bla_{\text{CTX-M-3}}$, $bla_{\text{SHV-12}}$, *qnrB*	I1	Yes	$bla_{\text{TEM-1}}$, $bla_{\text{CTX-M-3}}$, $bla_{\text{SHV-12}}$, *qnrB*
13	ST-BM1	*S.* Typhimurium	$bla_{\text{TEM-1}}$	A/C	No	-
14	ST-BM2	*S.* Typhimurium	$bla_{\text{TEM-1}}$, $bla_{\text{CTX-M-14}}$, $bla_{\text{OXA-1}}$, *qnrS*	HI2	Yes	$bla_{\text{TEM-1}}$, $bla_{\text{CTX-M-14}}$, *qnrS*
15	ST-BC1	*S.* Typhimurium	$bla_{\text{TEM-1}}$, $bla_{\text{CTX-M-13}}$, *qnrS*	HI1	Yes	$bla_{\text{TEM-1}}$, $bla_{\text{CTX-M-13}}$, *qnrS*
16	ST-BC2	*S.* Typhimurium	$bla_{\text{TEM-1}}$, *qnrA*	N	No	-
17	ST-BC3	*S.* Typhimurium	$bla_{\text{TEM-1}}$, *qnrS*	I1	Yes	$bla_{\text{TEM-1}}$, *qnrS*
18	SE-CM1	*S.* Enteritidis	$bla_{\text{TEM-1}}$, $bla_{\text{CTX-M-3}}$, $bla_{\text{CMY-2}}$, $bla_{\text{OXA-1}}$, *qnrB*, *aac(6′)-Ib-cr*	N	Yes	$bla_{\text{TEM-1}}$, $bla_{\text{CTX-M-3}}$, $bla_{\text{CMY-2}}$, *qnrB*, *aac(6′)-Ib-cr*
19	SE-CM2	*S.* Enteritidis	$bla_{\text{TEM-1}}$, $bla_{\text{CTX-M-15}}$, *qnrS*	A/C	Yes	$bla_{\text{TEM-1}}$, $bla_{\text{CTX-M-15}}$
20	SE-CM3	*S.* Enteritidis	$bla_{\text{TEM-1}}$, $bla_{\text{SHV-12}}$, *qnrS*	HI2	Yes	$bla_{\text{TEM-1}}$, $bla_{\text{SHV-12}}$, *qnrS*
21	SE-BC1	*S.* Enteritidis	$bla_{\text{OXA-1}}$, $bla_{\text{CMY-2}}$	N	No	-

22	SV-CM1	*S.* Virchow	$bla_{\text{TEM-1}}$, $bla_{\text{CTX-M-15}}$, *aac(6′)-Ib-cr*	A/C	Yes	$bla_{\text{TEM-1}}$, $bla_{\text{CTX-M-15}}$, *aac(6′)-Ib-cr*
23	SV-BM1	*S.* Virchow	$bla_{\text{TEM-1}}$, *qnrS*	N	Yes	$bla_{\text{TEM-1}}$, *qnrS*
24	SV-BC1	*S.* Virchow	$bla_{\text{TEM-1}}$, $bla_{\text{OXA-1}}$, $bla_{\text{CMY-2}}$	HI1	Yes	$bla_{\text{TEM-1}}$, $bla_{\text{CMY-2}}$
25	SV-BC2	*S.* Virchow	*qnrB*	I1	Yes	*qnrB*
26	SH-CM1	*S.* Heidelberg	$bla_{\text{TEM-1}}$, $bla_{\text{CTX-M-2}}$, $bla_{\text{CMY-2}}$, $bla_{\text{SHV-12}}$, *qnrB*	A/C	Yes	$bla_{\text{TEM-1}}$, $bla_{\text{CTX-M-2}}$, $bla_{\text{CMY-2}}$, $bla_{\text{SHV-12}}$, *qnrB*
27	SH-CM2	*S.* Heidelberg	$bla_{\text{TEM-1}}$, $bla_{\text{CMY-2}}$	I1	Yes	$bla_{\text{TEM-1}}$, $bla_{\text{CMY-2}}$
28	SH-CM3	*S.* Heidelberg	$bla_{\text{TEM-1}}$, *qnrB*	N	No	-
29	SK-CM1	*S.* Kentucky	$bla_{\text{TEM-1}}$, $bla_{\text{CTX-M-15}}$, $bla_{\text{CMY-2}}$, $bla_{\text{OXA-1}}$, *qnrS*, *aac(6′)-Ib-cr*	L/M	Yes	$bla_{\text{TEM-1}}$, $bla_{\text{CTX-M-15}}$, $bla_{\text{CMY-2}}$, *qnrS*, *aac(6′)-Ib-cr*
30	SK-CM2	*S.* Kentucky	$bla_{\text{TEM-1}}$, $bla_{\text{SHV-12}}$, *aac(6′)-Ib-cr*	I1	Yes	$bla_{\text{TEM-1}}$, $bla_{\text{SHV-12}}$, *aac(6′)-Ib-cr*
31	SAN-CM1	*S.* Anatum	$bla_{\text{OXA-1}}$, *qnrS*	A/C	Yes	*qnrS*
32	SAG-CM1	*S.* Agona	*qnrA*	N	No	-
33	SM-BC1	*S.* Montevideo	$bla_{\text{TEM-1}}$, *qnrS*	HI1	Yes	$bla_{\text{TEM-1}}$, *qnrS*
34	SS-BC1	*S.* Stanley	*qnrS*	HI2	Yes	*qnrS*

Table S6. Numbers and sources of meat samples used in this study

Products	No. samples	Source
1- Chicken meat		
a- Breast	80	street vendors, retail markets
b- Legs	80	street vendors, retail markets
Total	160	
2- Beef meat		
a- Frozen	80	street vendors, retail markets
b- Fresh	40	butchers
Total	120	
3- Beef carcass	120	swabs from slaughterhouses
Total	400	

Table S7. City names and numbers of meat samples collected from four governorates (Dakahlia, Damietta, Gharbia and Kafr El-Sheikh) in Egypt

**Dakahlia**		**Damietta**		Gharbia		Kafr El-Sheikh	
**City name**	**No.[a]**	**City name**	**No.[a]**	City name	No.[a]	City name	No.[a]
1. Aga	10	1. Al Zarqa	10	1.Al Santa	10	1. Baltim	10
2. Al **Manzalah**	10	2. Al Sarou	10	2. Basioun	10	2. Biyala	10
3. Belkas	10	3. Damietta	10	3. El Mahalla El Kubra	20	3. Desouk	10
4. Dekernes	10	4. El-Rawda	10	4. Kafr El-Zayat	10	4. El-Hamoul	10
5. El-Senbellawein	10	5. Faraskour	10	5. Qattour	10	5. El-Reyad	10
6. Mansoura	10	6. Izbat Al Borg	10	6. Samannoud	10	6. Fowah	10
7. Mataria	10	7. Kafr Al Battikh	10	7. Tanta	20	7. Kafr El Sheikh	10
8. Nabaruh	10	8. Kafr Saad	10	8. Zefta	10	8. Metoubes	10
9. Sherbin	10	9. Mit Abou Ghaleb	10	-	-	9. Qillin	10
10. Talkha	10	10. Ras Elbar	10	-	-	10. Sidi Salem	10
Total	100		100	Total	100	Total	100

[a] No., number of meat samples collected

Table S8. Primers used for PCR and DNA sequencing

Primer	Sequence (5' to 3')[a]	Annealing Temp.	Target	Reference
β-Lactamases				
TEM-F	ATAAAATTCTTGAAGACGAAA	50 °C	*bla*TEM	[50]
TEM-R	GACAGTTACCAATGCTTAATC			
SHV-F	TT ATCTCCCTGTTAGCCACC	50 °C	*bla*SHV	[50]
SHV-R	GATTTGCTGATTTCGCTCGG			
OXA-F	TCAACTTTCAAGATCGCA	56 °C	*bla*OXA	[50]
OXA-R	GTGTGTTTAGAATGGTGA			
CTX-M-F	CGCTTTGCGATGTGCAG	56 °C	*bla*CTX-M	[50]
CTX-M-R	ACCGCGATATCGTTGGT			
CMY-F	GACAGCCTCTTTCTCCACA	55 °C	*bla*CMY	[50]
CMY-R	TGGAACGAAGGCTACGTA			
Plasmid-mediated quiniolone				[50]
qnrA-F	ATTTCTCACGCCAGGATTTG	53 °C	*qnrA*	[50]
qnrA-R	GATCGGCAAAGGTTAGGTCA			
qnrB-F	GATCGTGAAAGCCAGAAAGG	53 °C	*qnrB*	[50]
qnrB-R	ACGATGCCTGGTAGTTGTCC			
qnrS-F	ACGACATTCGTCAACTGCAA	53 °C	*qnrS*	[50]
qnrS-R	TAAATTGGCACCCTGTAGGC			
aac(6')-Ib-F	TTGCGATGCTCTATGAGTGGCTA	55 °C	*aac(6′)-Ib-cr*	[50]
aac(6')-Ib-R	CTCGAATGCCTGGCGTGTTT			
Carbapenemases				
IMP-F	GGAATAGAGTGGCTTAAYTCTC	52 °C	*bla*IMP	[51]
IMP-R	GGTTTAAYAAAACAACCACC			
NDM-F	GGTTTGGCGATCTGGTTTTC	52 °C	*bla*NDM	[51]
NDM-R	CGGAATGGCTCATCACGATC			
SPM-F	AAAATCTGGGTACGCAAACG	52 °C	*bla*SPM	[51]
SPM-R	ACATTATCCGCTGGAACAGG			
VIM-F	GATGGTGTTTGGTCGCATA	52 °C	*bla*VIM	[51]
VIM-R	CGAATGCGCAGCACCAG			
OXA48-F	GCGTGGTTAAGGATGAACAC	52 °C	*bla*OXA-48	[51]
OXA48-R	CATCAAGTTCAACCCAACCG			
Plasmid-replicon typing				
A/C-F	GAGAACCAAAGACAAAGACCTGGA	60 °C	A/C	[52]
A/C-R	ACGACAAACCTGAATTGCCTCCTT			
B/O-F	GCGGTCCGGAAAGCCAGAAAAC	60 °C	B/O	[52]
B/O-R	TCTGCGTTCCGCCAAGTTCGA			
FIA-F	CCATGCTGGTTCTAGAGAAGGTG	52 °C	FIA	[52]
FIA-R	GTATATCCTTACTGGCTTCCGCAG			
FIC-F	GTGAACTGGCAGATGAGGAAGG	52 °C	FIC	[52]
FIC-R	TTCTCCTCGTCGCCAAACTAGAT			
FIIA/FIIS-F	CTGTCGTAAGCTGATGGC	52 °C	FIIA/FIIS	[52]
FIIA/FIIS-R	CTCTGCCACAAACTTCAGC			
FIB-F	GGAGTTCTGACACACGATTTTCTG	52 °C	FIB	[52]
FIB-R	CTCCCGTCGCTTCAGGGCATT			
HI1-F	GGAGCGATGGATTACTTCAGTAC	60 °C	HI1	[52]
HI1-R	TGCCGTTTCACCTCGTGAGTA			
HI2-F	TTTCTCCTGAGTCACCTGTTAACAC	60 °C	HI2	[52]
HI2-R	GGCTCACTACCGTTGTCATCCT			
I1-F	CGAAAGCCGGACGGCAGAA	60 °C	I1	[52]
I1-R	TCGTCGTTCCGCCAAGTTCGT			
K/B-F	GCGGTCCGGAAAGCCAGAAAAC	60 °C	K/B	[52]

K/B-R	TCTTTCACGAGCCCGCCAAA			
L/M-F	GGATGAAAACTATCAGCATCTGAAG	60 °C	L/M	[52]
L/M-R	CTGCAGGGGCGATTCTTTAGG			
N-F	GTCTAACGAGCTTACCGAAG	60 °C	N	[52]
N-R	GTTTCAACTCTGCCAAGTTC			
T-F	TTGGCCTGTTTGTGCCTAAACCAT	60 °C	T	[52]
T-R	CGTTGATTACACTTAGCTTTGGAC			
W-F	CCTAAGAACAACAAAGCCCCCG	60 °C	W	[52]
W-R	GGTGCGCGGCATAGAACCGT			
X-F	AACCTTAGAGGCTATTTAAGTTGCTGAT	60 °C	X	[52]
X-R	TGAGAGTCAATTTTTATCTCATGTTTTAGC			
